# Challenges in Diagnosis and Management of Unusual Cases of Eosinophilic Enteritis in Rural Health Settings: A Comprehensive Review

**DOI:** 10.7759/cureus.55398

**Published:** 2024-03-02

**Authors:** Shreya Ghosh, K. M. Hiwale

**Affiliations:** 1 Pathology, Jawaharlal Nehru Medical College, Datta Meghe Institute of Higher Education and Research, Wardha, IND

**Keywords:** global health impact, healthcare disparities, unusual cases, diagnosis challenges, rural health settings, eosinophilic enteritis

## Abstract

This comprehensive review delves into the challenges associated with diagnosing and managing unusual cases of eosinophilic enteritis in rural health settings. Eosinophilic enteritis, characterized by an abnormal accumulation of eosinophils in the gastrointestinal (GI) tract, poses distinct difficulties in diagnosis due to its varied presentations. In rural contexts, limited access to specialized diagnostic tools, a shortage of healthcare professionals, and geographical constraints compound these challenges. This abstract encapsulates the critical issues explored in the review, emphasizing the importance of addressing atypical cases and rural healthcare's unique hurdles. The conclusion is a rallying call for collaborative action, advocating for improved education, telemedicine solutions, and enhanced access to specialized care. The implications extend beyond eosinophilic enteritis, with the potential to instigate systemic improvements in rural healthcare globally. This review is a crucial contribution to understanding eosinophilic enteritis in rural settings and advocates for transformative measures to improve diagnosis, management, and overall healthcare outcomes.

## Introduction and background

Eosinophilic enteritis is characterized by an abnormal accumulation of eosinophils, a type of white blood cell, in the lining of the gastrointestinal (GI) tract. While the condition can affect any part of the digestive system, its manifestations often vary, leading to difficulties in diagnosis. Understanding the fundamental characteristics of eosinophilic enteritis is crucial for healthcare professionals aiming to address its diverse presentations [1]. Unusual or atypical presentations of eosinophilic enteritis can complicate the diagnostic process, leading to delayed treatment and increased morbidity. Recognizing the importance of investigating these uncommon cases is pivotal for enhancing our understanding of the disease spectrum and improving patient outcomes [2].

Rural health settings present unique challenges in diagnosing and managing medical conditions, including eosinophilic enteritis. Limited access to specialized healthcare resources, a shortage of experienced professionals, and geographical constraints can contribute to delayed interventions. This review focuses on the difficulties faced in rural areas, emphasizing the need for tailored approaches to effectively address eosinophilic enteritis [3]. This review aims to provide a comprehensive examination of the challenges associated with unusual cases of eosinophilic enteritis, specifically within rural health settings. By exploring diagnostic hurdles, management strategies, and the impact on patients and communities, this review aims to contribute valuable insights for healthcare professionals, researchers, and policymakers. The ultimate goal is to foster a better understanding of eosinophilic enteritis in rural contexts and propose measures to improve diagnosis and patient care.

## Review

Background

Definition and Classification of Eosinophilic Enteritis

Eosinophilic enteritis represents a rare and heterogeneous spectrum of disorders characterized by eosinophilic infiltration across various organs and GI tract layers. It falls within the broader category of eosinophilic gastrointestinal disorders (EGIDs), which are further categorized into three subtypes (mucosal, muscular, and serosal) based on the specific layer of the GI tract affected by eosinophilic infiltration [4]. Diagnosing eosinophilic enteritis typically involves GI symptoms and histological evidence of eosinophilic infiltration in one or more GI tract areas [4]. The disease exhibits a widely variable and heterogeneous profile of physical manifestations, with a prevalence ranging between 8.4 and 28 per 100,000, displaying a slightly increasing incidence over the past five decades [4,5]. Although the molecular mechanisms underlying this condition remain elusive, a predominant role of hypersensitivity responses in its pathogenesis is evident, with many patients having a history of seasonal allergies, food sensitivities, asthma, and eczema [6]. The prevalence of eosinophilic gastroenteritis (EGE) has notably increased, likely attributed to historical under-diagnosis [5]. Eosinophilic enteritis can affect individuals of all ages, with a peak prevalence observed in those aged 20-50 years [5]. Diagnosis relies on the histological examination of gastric and duodenal biopsies, often necessitating multiple specimens to mitigate the risk of sampling error and ensure accurate diagnosis [6]. This chronic and relapsing disease, with spontaneous remission reported in around 30%-40% of cases, generally requires ongoing treatment [6].

Prevalence and Incidence in Rural Areas

Eosinophilic esophagitis (EoE): Studies have indicated a noteworthy association between EoE and geographic factors, particularly in rural areas with low population density. A specific investigation discovered a higher prevalence of esophagal eosinophilia in rural regions [7]. Furthermore, another study focused on children revealed a significantly elevated occurrence of EoE in rural areas compared to their urban counterparts [8]. A comprehensive meta-analysis provided an overall pooled prevalence of EoE at 22.7 per 100,000 population, with a more pronounced rate observed in adults (43.4 per 100,000) [9]. These findings highlight the potential influence of rural environments on the prevalence of EoE and underscore the need for further research to elucidate the underlying factors contributing to this geographic variation.

Eosinophilic colitis: Research utilizing the National Inpatient Sample (NIS) database identified 4,353 cases of eosinophilic colitis, revealing that a minority (16.5%) of these cases were reported in rural areas [10]. Furthermore, an analysis of hospitalization patterns demonstrated a substantial concentration of eosinophilic colitis cases in urban teaching hospitals (77.8%) compared to urban nonteaching hospitals (17.6%) [10]. This data suggests a notable urban predominance in the hospitalization of eosinophilic colitis cases. However, the relatively low representation of rural cases prompts further investigation into the epidemiology and distribution of eosinophilic colitis in rural settings.

Hospitalization patterns: Examining the hospitalization data for eosinophilic colitis, a clear pattern emerges where most admissions occur in urban teaching hospitals (77.8%). In contrast, a significantly lower percentage (17.6%) of admissions was recorded in urban nonteaching hospitals [10]. This discrepancy in hospitalization patterns indicates potential variations in access to healthcare resources and expertise between different healthcare facilities in urban areas. While these studies contribute valuable insights into the epidemiology of eosinophilic enteritis, particularly about hospitalization, there remains a critical need for more extensive research to comprehensively establish the prevalence and incidence of these disorders, specifically in rural settings.

Factors Contributing to Challenges in Diagnosis and Management

Heterogeneity of the disease: Eosinophilic enteritis is a rare and intricate group of disorders marked by an eosinophilic infiltrate across various organs and layers of the GI tract [4]. The disease's protean manifestations, contingent upon the specific involvement of intestinal segments and layers, pose a formidable challenge for clinicians attempting an accurate diagnosis [4]. This inherent heterogeneity complicates the understanding and classification of the disease, necessitating a nuanced approach to diagnosis and management.

Differential diagnosis: Eosinophilic enteritis introduces a diagnostic challenge due to its potential confusion with conditions such as irritable bowel syndrome or parasitic infections [4]. A comprehensive and broad differential diagnosis becomes imperative, particularly considering atypical presentations that may mimic infectious pathogens [11]. A meticulous approach to ruling out alternative causes is crucial for precise identification and subsequent management.

Environmental factors: Influential environmental factors, including antibiotic use and dietary choices, exert a discernible impact on the normal levels of GI eosinophils [4]. In rural areas, where these factors might be more prevalent, attributing eosinophilia to a specific cause becomes notably intricate. The intricate interplay between environmental influences and eosinophilic enteritis underscores the complexity of unraveling its etiology.

Malnutrition: Persistent GI inflammation and abnormal intestinal absorption in eosinophilic enteritis can lead to malnutrition among affected patients [4]. This nutritional consequence not only exacerbates the challenges associated with diagnosis but also presents an additional layer of complexity in managing these disorders. Addressing malnutrition becomes integral to the comprehensive care and treatment of individuals with eosinophilic enteritis.

Health disparities: Disparities in the presentation and care of eosinophilic enteritis, particularly in pediatric cases, have been linked to factors such as racial background, socioeconomic status, and other determinants [4]. These disparities may be further exacerbated in rural health settings, where access to specialized care and resources may be limited. Recognizing and addressing these disparities is crucial for ensuring equitable and effective healthcare delivery.

Limited resources: Rural health settings often need more resources and expertise to effectively diagnose and manage eosinophilic enteritis [4]. This limitation contributes to potential delays in diagnosis and suboptimal treatment, adversely impacting patient outcomes. The scarcity of specialized resources in rural areas necessitates strategic interventions to bridge this gap and enhance the overall quality of care for individuals with eosinophilic enteritis.

Clinical presentation

Common Symptoms

The clinical presentation of eosinophilic enteritis exhibits variability contingent upon the location and severity of eosinophilic infiltration within the GI tract. Predominant symptoms encompass abdominal pain, nausea, vomiting, diarrhea, weight loss, abdominal distention, and dysphagia [12]. Peripheral eosinophilia and ascites may additionally manifest in some instances [11]. The duration of symptoms emerges as a potential risk factor for EGE [12]. It is crucial to acknowledge that eosinophilic enteritis poses a diagnostic challenge, often susceptible to being overlooked or misidentified as other conditions like chronic gastritis or non-specific enteritis [12]. The imperative for a comprehensive differential diagnosis becomes apparent, particularly in considering atypical presentations that might mimic infectious pathogens [11]. Disparities in the presentation and management of eosinophilic enteritis among pediatric cases have been linked to factors such as racial background, socioeconomic status, and other determinants [13]. This underscores the necessity for heightened awareness and tailored approaches to ensure equitable care for all affected individuals.

Unusual or Atypical Presentations

Eosinophilic enteritis, an uncommon condition with an elusive etiology, manifests with atypical symptoms and unconventional presentations. Typically emerging during childhood and early adolescence, it predominantly impacts the stomach and small bowel [14]. Remarkably, EGE can exhibit diverse and uncommon presentations, encompassing gastric outlet and small bowel obstruction, esophagitis, gastritis featuring polypoid lesions, ulcers, or erosions, colitis, pancreatitis, and even masquerading as malignancy [2]. Furthermore, it may manifest as an acute abdomen, precipitated by acute pancreatitis, intestinal or colonic obstruction, intussusception, or perforation [2]. These varied and atypical presentations underscore the intricate nature of diagnosing eosinophilic enteritis, underscoring the imperative for heightened clinical suspicion and a comprehensive differential diagnosis. The challenge lies in recognizing the myriad ways this condition may present, necessitating a vigilant and inclusive diagnostic approach to ensure accurate identification and appropriate management.

Case Studies Highlighting Unique Challenges in Rural Settings

Eosinophilic enteritis and other EGIDs pose distinctive challenges in rural health settings, significantly impacting the prompt diagnosis and effective management of these conditions. Various factors contribute to these challenges, including limited access to specialized healthcare, environmental influences, and health disparities. A study focusing on the impediments to the timely diagnosis of EGIDs specifically emphasized the adverse effects of rural environments on addressing feeding difficulties in patients with inflammatory bowel disease (IBD) [15]. Notably, a perspective originating from sub-Saharan Africa highlighted structural deficiencies within healthcare systems, inadequate endoscopic capacity, and limited access to diagnostic imaging as formidable challenges in the timely and accurate diagnosis of IBD, illustrating their relevance to the diagnosis of EGIDs in rural areas [16]. These findings underscore the imperative for heightened awareness, improved access to specialized care, and enhanced diagnostic capabilities in rural health settings. Addressing these unique challenges is pivotal for ensuring timely, accurate diagnoses and effective management of eosinophilic enteritis and other EGIDs in rural communities.

Diagnostic challenges

Limited Access to Specialized Diagnostic Tools

Lack of access to endoscopy and biopsy: The diagnostic process for eosinophilic enteritis often necessitates endoscopic procedures and biopsies. However, rural areas frequently encounter challenges associated with limited access to these specialized diagnostic tools. Resources need to be improved to ensure the timely obtaining of a definitive diagnosis, facilitating the timely initiation of appropriate treatment [4]. The crucial role of endoscopy and biopsies in confirming eosinophilic enteritis underscores the significance of addressing these access limitations in rural healthcare settings.

Diagnostic disparities among children: Research indicates that children residing in rural areas may face diagnostic disparities concerning eosinophilic enteritis [17]. This limitation affects the accuracy and timeliness of diagnosis in pediatric cases. It underscores the need for targeted interventions to bridge the gap in diagnostic resources between rural and urban healthcare settings.

Limited disease awareness and guidelines: The absence of widespread disease awareness and specific diagnostic guidelines for eosinophilic enteritis poses a significant challenge, particularly in settings with limited access to specialized care [18]. This knowledge gap may lead to patients needing to be diagnosed or experiencing diagnostic delays, emphasizing the critical need for comprehensive education initiatives and the development of clear diagnostic protocols to improve the recognition and management of eosinophilic enteritis in rural and urban healthcare environments.

Histopathological examination requirements: The confirmation of eosinophilic enteritis hinges on histopathological examination of biopsies, which must demonstrate a substantial eosinophil count per high-power field. However, lacking access to facilities equipped for these specialized examinations can contribute to diagnostic delays [19-20]. Overcoming this challenge requires strategic efforts to enhance the availability of histopathological examination resources in rural health settings, ensuring that patients receive timely and accurate diagnoses essential for appropriate medical interventions.

Lack of Awareness Among Healthcare Professionals

The notable absence of awareness among healthcare professionals regarding eosinophilic enteritis poses a substantial challenge, as evidenced by a study revealing a predominant concentration of patients admitted for eosinophilic colitis in urban teaching hospitals. This observation suggests a potential need for more awareness in non-teaching hospitals and rural settings [10]. Additionally, the escalating incidence of patients with incidental eosinophilia in clinical practice underscores the persistent challenge of diagnosing and effectively treating these conditions [21]. Rectifying this lack of awareness necessitates concerted efforts in education and the formulation of comprehensive diagnostic guidelines. This imperative is crucial for enhancing the timely and accurate diagnosis of eosinophilic enteritis, particularly in rural health settings.

Overlapping Symptoms With Other GI Disorders

Eosinophilic enteritis often manifests with symptoms that are non-specific and may overlap with those of other GI disorders, posing a considerable diagnostic challenge. Common symptoms associated with eosinophilic enteritis encompass nausea, vomiting, diarrhea, abdominal pain, bloating, and weight loss [22-24]. A comprehensive differential diagnosis must consider atypical presentations that mimic infectious pathogens, irritable bowel syndrome, or parasitic infections [22,23]. Environmental factors, such as antibiotic use and dietary habits, can impact the normal levels of GI eosinophils, and these influences may be more prevalent in rural areas, complicating the determination of the cause of eosinophilia [22,25,26]. To address these challenges effectively, healthcare providers in rural settings must be vigilant about potential confounders and recognize the atypical presentations of eosinophilic enteritis. Collaborating with specialists and referring patients to tertiary care centers when necessary is crucial to ensure a proper diagnosis and facilitate appropriate management. This collaborative approach is vital for optimizing patient outcomes in the context of eosinophilic enteritis within rural healthcare settings.

Management strategies

Review of Current Treatment Options

Dietary restrictions: A prevalent treatment strategy for EoE and EGE involves the elimination of foods triggering symptoms. This approach often utilizes food allergy testing to pinpoint problematic foods, leading to their removal from the patient's diet [27]. By identifying and excluding specific dietary triggers, healthcare providers aim to alleviate symptoms and mitigate the inflammatory response within the GI tract.

Systemic corticosteroids or anti-inflammatory drugs: Medications like systemic corticosteroids or other anti-inflammatory drugs are pivotal in managing EoE and EGE by mitigating pain, swelling, and redness in the GI tract. Examples include ibuprofen or naproxen sodium, which aim to alleviate the inflammatory burden on the digestive system [27]. These medications contribute to symptom relief and are instrumental in controlling the overall inflammatory response within the GI tract.

Proton pump inhibitors (PPIs): PPIs are employed in treating EGE by reducing acid production in the stomach [28]. This approach aims to modulate the acidic environment within the GI tract, addressing one of the contributing factors to inflammation. PPIs are thus incorporated into the treatment plan to alleviate symptoms and promote overall GI health.

Leukotriene modifiers: Leukotriene modifiers emerge as medications capable of reducing inflammation and improving symptoms in certain patients with EoE [4]. By targeting specific pathways in the inflammatory cascade, these modifiers ameliorate the inflammatory response within the GI tract, contributing to overall symptom management.

Mast cell stabilizers: Mast cell stabilizers serve as drugs designed to dampen the activity of mast cells, pivotal players in the inflammatory response in EoE [4]. These medications aim to curb the inflammatory cascade within the GI tract by reducing mast cell activity, addressing one of the critical mechanisms contributing to the condition.

Biologic therapies: For specific individuals with EoE, biologic therapies represent a valuable treatment option. These therapies target specific molecules involved in the inflammatory response, such as interleukin-5 (IL-5) or interleukin-13 (IL-13) [28]. By precisely modulating these inflammatory pathways, biological therapies offer a targeted and practical approach to managing the underlying causes of EoE.

Endoscopic and histological monitoring: Regular endoscopies and biopsies form a crucial component of the treatment approach, enabling ongoing monitoring of the patient's condition and assessment of treatment effectiveness [29]. These procedures are essential for healthcare providers to make informed decisions regarding treatment adjustments and ensure that the chosen therapeutic interventions yield the desired outcomes. The treatment option is tailored to each patient's symptoms, disease severity, and individual medical conditions, with healthcare providers carefully considering these factors to develop a personalized and effective treatment plan for each patient [27].

Challenges in Administering Standard Therapies in Rural Areas

Limited access to healthcare services: Rural areas often grapple with constrained access to healthcare services, particularly specialized care, creating challenges for patients with EoE and EGE in receiving the requisite treatment [30]. The scarcity of accessible healthcare services can significantly impede the timely and appropriate care for individuals with these conditions.

Workforce shortages: Workforce shortages in rural healthcare settings pose a persistent challenge, making attracting and retaining staff difficult. This situation leads to burnout among existing healthcare providers and exacerbates the scarcity of healthcare services in rural areas [30]. The impact of staff shortages reverberates through the healthcare system, hindering the delivery of essential services, including those required to diagnose and manage EoE and EGE.

Lack of training: Healthcare providers in rural areas may need specialized training for diagnosing and managing conditions like EoE and EGE. This inadequate training can delay diagnosis and suboptimal patient treatment [31]. The absence of specialized knowledge contributes to challenges in addressing the specific needs of individuals grappling with these GI disorders in rural healthcare settings.

Barriers to telehealth: While telehealth holds promise for delivering healthcare services in rural areas, obstacles such as insufficient internet infrastructure and patients' need to travel long distances to access specialty providers can limit its effectiveness [32]. Overcoming these barriers is crucial for leveraging telehealth as a viable solution. Initiatives to improve healthcare access in rural areas encompass financial incentives for rural practice, specialized training tailored to rural-specific challenges, and infrastructural enhancements, including broadband access [30]. Additionally, expanding telehealth capabilities can enhance access to care, contingent on addressing and mitigating the barriers specific to its utilization in rural settings [32].

Potential of Telemedicine and Remote Consultation

Telemedicine and remote consultation present a promising avenue for enhancing access to specialized care, particularly for patients grappling with GI disorders such as IBD and eosinophilic enteritis, especially those residing in rural areas [33-35]. The utilization of telemedicine has shown potential not only to improve access but also to enhance patient satisfaction and reduce post-discharge testing rates [33]. However, it remains imperative to critically evaluate whether this approach outperforms established and more cost-effective alternatives [33]. Telemedicine has applications across various aspects of patient care, encompassing previsit triage, urgent care, routine follow-up, and medication adherence [35,36]. Its implementation can potentially mitigate the necessity for hospitalization, thereby contributing to improved patient outcomes [33,35]. In GI disorders, a multidisciplinary care approach involving collaborative efforts from diverse healthcare professionals can provide holistic and comprehensive care. This may include diagnostic testing, educational initiatives, and support for the patient and their family [37]. The versatility of telemedicine is evident in its applicability to different modes of follow-up care, including phone consultations, in-person appointments, and, under specific circumstances, remote consultations [37]. Leveraging telemedicine in the continuum of care for patients with GI disorders holds promise for optimizing resource utilization, enhancing accessibility, and improving the overall quality of care. As this technology continues to evolve, ongoing assessment of its effectiveness, cost-efficiency, and patient satisfaction is paramount to ensure its integration complements and enhances existing healthcare practices.

Case studies

Detailed Analysis of Select Unusual Cases in Rural Health Settings

Eosinophilic enteritis flare-up resembling acute gastroenteritis: A 33-year-old woman hailing from Vietnam presented with sudden abdominal pain, newly occurring eosinophilic ascites, peripheral eosinophilia, and no identifiable risk factors for parasitic infections [11]. Initially misdiagnosed with acute gastroenteritis, a more in-depth investigation uncovered EGE as the underlying cause. Treatment involving ivermectin and mebendazole resulted in a complete resolution of her symptoms [11]. This case underscores the significance of adopting a comprehensive approach to the differential diagnosis of eosinophilic ascites, considering atypical presentations of infectious pathogens [11].

EGE in a child's small intestine: In a remote medical center in the Middle East and Levantine region, a child was diagnosed with EGE affecting the small intestine [38]. Pathological assessment led to the diagnosis, and the subsequent treatment required surgical intervention. This case emphasizes the critical need for communication among medical centers in remote areas, facilitating discussions about atypical cases with seasoned professionals [38]. Both instances illustrate the complexities of diagnosing and managing eosinophilic enteritis in rural health settings. Healthcare providers in these contexts must be attuned to potential confounders and the atypical presentations of eosinophilic enteritis. Collaborative efforts with specialists and timely referrals to tertiary care centers are essential to ensure accurate diagnosis and effective management. Additionally, concerted endeavors to enhance access to specialized care and resources in rural areas are imperative, aiming to diminish health disparities and enhance patient outcomes.

## Conclusions

In conclusion, the review of unusual cases of eosinophilic enteritis in rural health settings underscores the intricate challenges inherent in diagnosing and managing this inflammatory disorder. The recapitulation of key challenges reveals the multifaceted nature of the impediments faced in rural areas, ranging from limited access to specialized diagnostic tools to the prevalence of atypical presentations. However, the concluding section is not merely a reflection but a call to action. It urges healthcare professionals, policymakers, and communities to unite in addressing these challenges head-on. By investing in education, implementing telemedicine solutions, and improving access to specialized care, a concerted effort can be made to enhance the diagnosis and management of eosinophilic enteritis in rural health settings. Significantly, the implications extend beyond this specific condition, holding the potential to catalyze systemic improvements in rural healthcare. Recognizing the interconnected nature of healthcare challenges globally, the review advocates for a holistic approach that considers the broader impact on rural healthcare systems and the potential contribution to addressing similar issues on a global scale. Thus, the conclusion serves not only as a summary of challenges but as a pivotal moment for catalyzing positive change in the landscape of rural healthcare and its potential reverberations on a global health stage.
